# Fibronectin glomerulopathy complicated with persistent cloaca and congenital esophageal atresia: a case report and literature review

**DOI:** 10.1186/s12882-017-0704-5

**Published:** 2017-09-06

**Authors:** Misaki Takii, Takaichi Suehiro, Aya Shima, Hideki Yotsueda, Satoshi Hisano, Ritsuko Katafuchi

**Affiliations:** 1 Department of Nephrology, Harasanshin Hospital, Fukuoka, Japan; 2 Department of Internal Medicine, Social Insurance Nakabaru Hospital, 2-12-1 Befukita, Shimemachi, Kasuya-gun, Fukuoka, 811-2233 Japan; 3 Department of Pathology, Faculty of Medicine, Fukuoka University, Fukuoka, Japan; 4 Kidney Unit, National Fukuoka-Higashi Medical Center, Fukuoka, Japan

**Keywords:** Fibronectin glomerulopathy, Fibronectin 1 gene, Congenital esophageal atresia, Persistent cloaca

## Abstract

**Background:**

Fibronectin glomerulopathy is a rare, inherited, autosomal dominant, glomerular disease characterized by proteinuria, microscopic hematuria, hypertension, massive glomerular deposits of fibronectin, and slow progression to end-stage renal failure. Because the incident of fibronectin glomerulopathy is extremely low, the pathophysiology, genetic abnormalities, epidemiology, and mechanisms remain to be elucidated.

**Case presentation:**

We report a 21-year-old woman with fibronectin glomerulopathy, who had been diagnosed with persistent cloaca and congenital esophageal atresia at birth. She developed proteinuria and hematuria 7 months before admission. Urinary protein and serum creatinine levels were 3.38 g/gCr and 0.73 mg/dL. Renal biopsy showed severe mesangial widening due to massive deposits, which was positive periodic acid-Schiff and negative methenamine silver. Immunostaining was negative for immunoglobulin but positive for fibronectin. Electron microscopy showed diffuse mesangial granular deposits. Thus she was diagnosed with fibronectin glomerulopathy, despite a negative family history of kidney disease and lack of any known missense mutations of fibronectin 1 gene.

**Conclusion:**

We report a patient who developed fibronectin glomerulopathy during the clinical course of extremely rare congenital malformations, including persistent cloaca and congenital esophageal atresia. We describe a case of this condition in detail and summarize the 75 case reports of fibronectin glomerulopathy.

## Background

Fibronectin glomerulopathy is a rare, inherited, autosomal dominant, glomerular disease characterized by proteinuria, microscopic hematuria, hypertension, massive glomerular deposits of fibronectin, and slow progression to end-stage renal failure (ESRF) [1]. Three heterozygous missense mutations in the fibronectin 1 gene (*FN1*) associated with fibronectin glomerulopathy were first reported by Castelletti et al. in 2008 [2]. However, the pathophysiology, genetic abnormalities, epidemiology, and mechanisms of fibronectin glomerulopathy remain to be elucidated. Persistent cloaca and congenital esophageal atresia are extremely rare congenital malformations, sometimes associated with the development of renal insufficiency [3, 4]. Here, we report a case of fibronectin glomerulopathy coexisting with congenital malformations, including persistent cloaca and congenital esophageal atresia.

## Case presentation

A 21-year-old Japanese woman was admitted to our hospital because of proteinuria and hematuria. She had been diagnosed at birth with persistent cloaca and congenital esophageal atresia, and underwent enterostomy, cystostomy, and radical esophageal surgery, followed by repair of persistent cloaca and colostomy closure 2 years later. She was followed-up at the pediatric surgery and urology department, and developed with no major problems. Proteinuria and hematuria were detected at routine examination 7 months prior to admission for the first time. No urological problems were detected at a visit to the urology department 3 months before admission. She had mild edema before admission; however its onset had been gradual and it was therefore not clear when the edema had started.

On admission, the patient’s height was 158.6 cm, her weight was 53.0 kg, and her blood pressure was 101/80 mmHg. Physical examination revealed an abdominal midline operation scar and pretibial pitting edema. Urinary examination revealed a protein level of 3+ and 5–9 red blood cells per high-power field. Her urine protein/creatinine ratio was 3.38 g/gCr, blood urea nitrogen was 13.2 mg/dL, serum creatinine was 0.73 mg/dL, and serum albumin was 3.6 g/dL. Serum C3 was 25 mg/dL, C4 was 18.5 mg/dL, and 50% hemolytic unit of complement (CH50) was 13 U/mL. Chromosome G banding revealed a normal karyotype 46XX. Kidney ultrasound showed no kidney deformity. Kidneys size was normal.

**Fig. 1:**
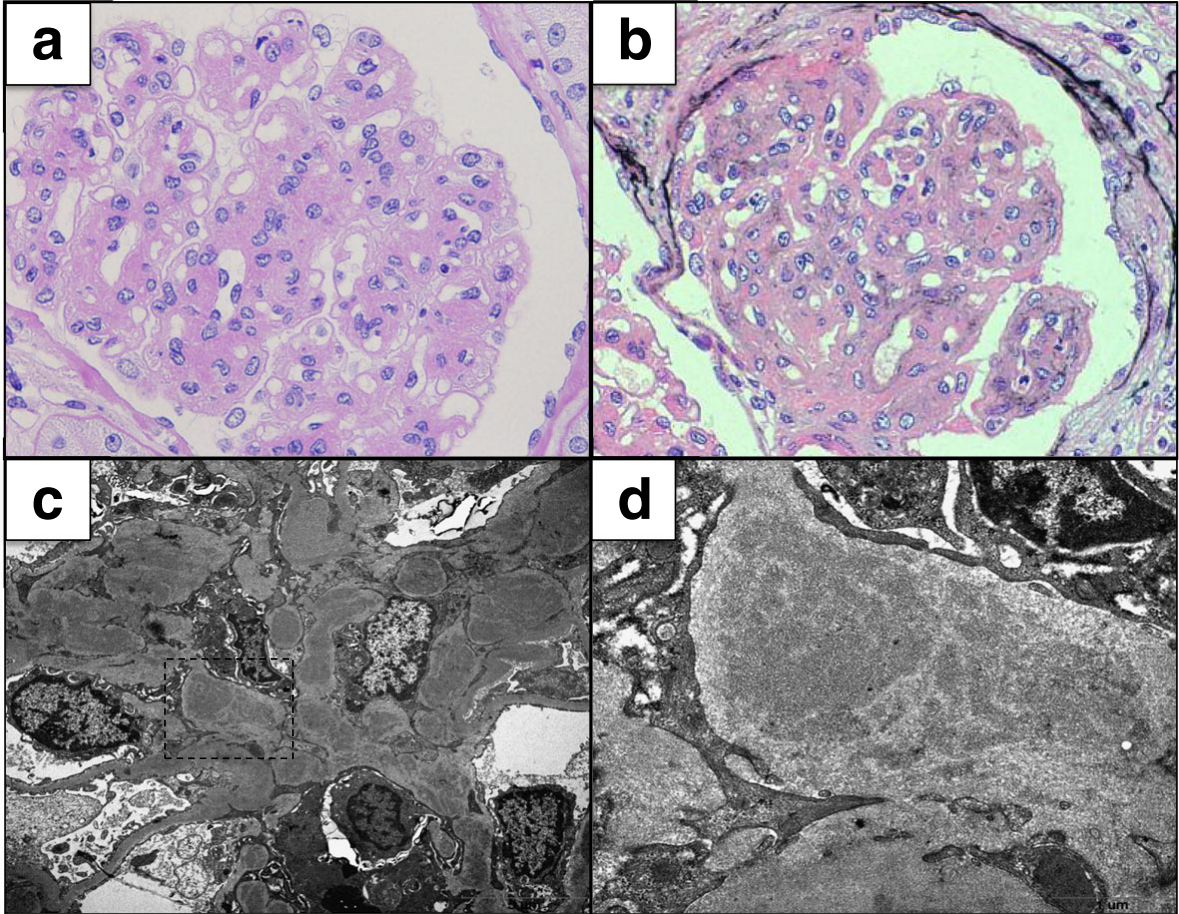
Light and electron microscopic images of kidney biopsy sections. **a** Periodic acid-Schiff (PAS) stain. A glomerulus showing mesangial hypercellularity and increased in PAS-positive materials in the mesangium. Original magnification, ×400. **b** Periodic acid-methenamine-silver stain. Increased materials in the mesangium were negative for methenamine-silver staining. Original magnification, ×200. **c**, **d** Electron microscopy showed diffuse granular deposits in the mesangium and the subepithelial spaces of the glomerular basement membrane. Original magnification, ×4000 (**c**) and ×10,000 (**d**)

**Fig. 2:**
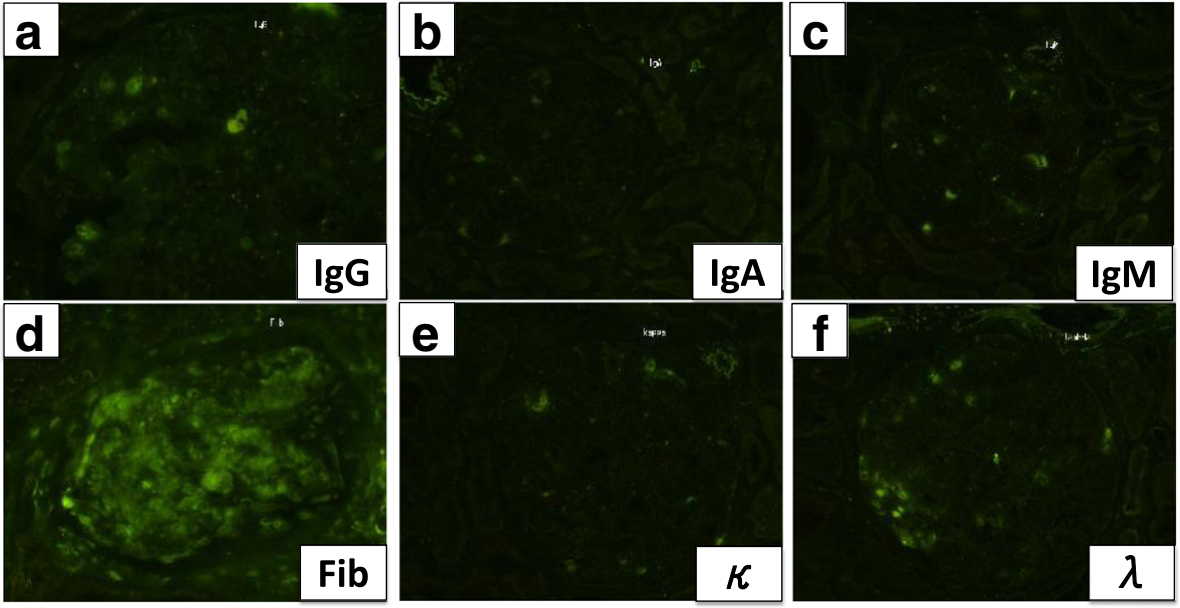
Immunofluorescent staining. Immunofluorescent staining revealed slight staining for fibrinogen (**d**), but no staining for IgG, IgA, IgM, kappa light chain and lambda light chain (**a**-**c**, **e**, **f**)

**Fig. 3:**
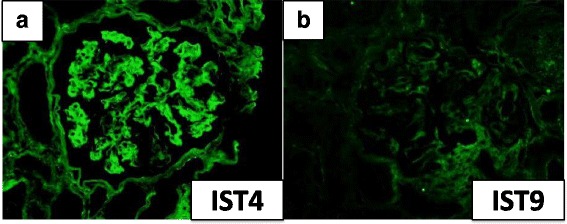
Immunohistochemical staining for fibronectin. **a** Staining with IST-4 showed a positive mesangial pattern. **b** Staining with IST-9 was negative

A renal biopsy contained 24 glomeruli, all of which showed moderate to severe mesangial hypercellularity and increased homogenous materials in the mesangium. Which were positive with periodic acid-Schiff and were negative with methenamine silver (Fig. 1a, b). One glomerulus revealed fibrous crescent formation, and 11 showed segmental double contour of glomerular basement membrane. Immunofluorescence microscopy showed slight staining for fibrinogen, but no staining for IgG, IgA, IgM, kappa light chain and lambda light chain (Fig. 2). Electron microscopy showed massive granular deposits in the mesangial area and some subepithelial area (Fig. 1c, d). According to these findings, we speculated fibronectin glomerulopathy as a diagnosis. Thus, we performed immunoassays using anti-human fibronectin antibodies. The staining with IST-4, which detects plasma and cell-associated fibronectin, was positive but no staining was observed with IST-9, which stains only cell-associated fibronectin (Fig. 3). The mesangial deposits were therefore shown to comprise plasma fibronectin. The patient was finally diagnosed as fibronectin glomerulopathy.

Her family history was investigated further. Although her mother and younger sister had previously been detected to have proteinuria, their urine and blood tests were normal. Her father showed no urinary abnormalities and normal kidney function at his annual health examination. Given that no family history of renal disease could be identified, the current case was determined to be sporadic. The patient and her mother underwent genetic testing of the *FN1* gene, which revealed no abnormalities.

Treatment with an angiotensin II receptor antagonist and a sodium-restricted diet were started. However, 10 months after the kidney biopsy, the patient’s proteinuria had increased and her renal function was deteriorated, with a urine protein/creatinine ratio of 9.52 g/gCr, serum creatinine 0.86 mg/dL. Her serum albumin decreased to 2.0 g/dL.

## Discussion

We herein present a patient who developed fibronectin glomerulopathy during the clinical course of rare congenital malformations, including persistent cloaca and congenital esophageal atresia. Notably, this case was sporadic, and showed no detectable *FN1* mutations.

Fibronectin glomerulopathy is a rare, inherited, autosomal dominant, glomerular disease characterized by massive fibronectin deposits in the glomeruli. It was first recognized as a distinct clinical entity by Strøm et al. in 1995 [1]. Common clinical features are mild to nephrotic range proteinuria, microscopic hematuria, hypertension, and deterioration of renal function [1, 5]. The disease often progresses to ESRF within 15–20 years from clinical onset [5, 6]. Fibronectin glomerulopathy is usually diagnosed between the second and fifth decades of life [6], but the disease can manifest at any age, with the youngest patient diagnosed at 3 years old [7] and the oldest at 78 years old [5]. Diagnosis can only be made by renal biopsy, because the clinical features are non-specific [1, 5]. Generally, light microscopy shows enlarged glomeruli with minimal hypercellularity, with extensive deposits in the mesangium and subendothelial spaces. Electron microscopy shows finely granular or fibrillary mesangial and subendothelial electron-dense deposits [1], with granular deposits revealing a fibrillary substructure at higher magnifications, with randomly arranged fibrils 12–14 nm wide [5]. The deposits in fibronectin glomerulopathy are non-immunoreactive by routine immunofluorescence microscopy, and an accurate diagnosis is accomplished by immunofluorescent staining for fibronectin [6]. In immunolocalization studies, fibrillary deposits were shown to be positive with IST-4, which is an antibody that detects both plasma fibronectin and cell-associated fibronectin, but were only weakly stained with IST-9, which is specific for cell-associated fibronectin, suggesting that the fibrils were deposits of plasma fibronectin [8].

The current case was notable for the presence of rare congenital malformations. Persistent cloaca is an extremely rare congenital malformation, occurring in only one in 50,000 live births. It is characterized by a single perineal opening for the urinary, gastrointestinal, and reproductive tracts [9]. Eighty percent of patients with persistent cloaca are also born with a structural abnormality of the urinary tract, including renal dysplasia, ectopic kidney, solitary kidney, duplex kidneys, and ureteropelvic junction obstruction [3]. Vesicoureteral reflux occurs in 41%–57% of persistent cloaca cases, and sacral abnormalities are diagnosed in 57%. About half of all patients with persistent cloaca develop chronic renal failure, with a glomerular filtration rate of <80 mL/min/1.73m^2^, and 17% progress to ESRF [3, 10]. Congenital esophageal atresia is also a relatively rare congenital malformation, occurring in one in 4000 live births [11]. It is a developmental defect of the foregut, characterized lack of continuity of the esophagus [4]. There have been few reports of renal disease associated with congenital esophageal atresia. Patients displaying the VATER association, which includes vertebral defects, anal atresia, trachea-esophageal fistula with esophageal atresia, and radial and renal dysplasia, often develop chronic renal failure [12], and congenital nephrotic syndrome [13]. Renal abnormality with 17q12 duplication have also been reported in patients with esophageal atresia [14]. However, as far as we investigated there has been no case report of fibronectin glomerulopathy complicated with these congenital malformations.

Seventy-five cases of fibronectin glomerulopathy have been reported, so far with full clinical data (Table 1) [1, 2, 5–8, 15–22]. Proteinuria was recorded in 69 cases, of which 35 had nephrotic-range proteinuria (>3.5 g/24 h). Twenty-two cases had proteinuria and serum albumin, of which 15 met the definition of nephrotic syndrome (proteinuria >3.5 g/24 h and serum albumin <3.0 g/dl).

**Table 1:** Clinical characteristics of the 75 reported cases of fibronectin glomerulopathy

		Age	Sex	UP	Cre	Comorbidity	Family history	FN1 mutation
1	Strøm et al.	59	F	3.9	1.0	NA	+	NA
2		18	M	4.5	1.2	NA	+	NA
3		24	M	14.0	1.0	NA	+	NA
4		21	M	2–4	1.0	NA	+	NA
5		19	M	NA	NA	NA	+	NA
6		22	F	NA	NA	NA	+	NA
7		25	F	5.0	NA	NA	+	NA
8		14	F	NS	0.6	NA	+	NA
9		64	M	2.0	1.1	NA	+	NA
10		16	M	5.0	NA	NA	+	NA
11		23	M	0.6	0.8	NA	+	NA
12		25	F	0.3	0.8	NA	+	NA
13		30	M	2.1	1.3	NA	+	NA
14		24	M	1.3	1.4	NA	+	NA
15		26	F	+	NA	cerebrovascular hemorrhage	+	NA
16		29	M	3+	1.8	cerebrovascular hemorrhage	+	NA
17		15	M	4+	NA	cerebrovascular hemorrhage myocardial infarction	+	NA
18		14	M	3+	0.8	NA	+	NA
19		44	M	4.7	1.6	NA	+	NA
20		37	F	0.7	0.9	NA	+	NA
21		33	M	1.0	1.0	NA	+	NA
22		31	M	2.1	1.0	NA	+	NA
23		53	M	NS	NA	renal cell carcinoma	+	NA
24	Gemperle et al.	35	F	5.6	NA	NA	+	NA
25		31	M	1.8	NA	myocardial infarction	+	NA
26		38	M	+	NA	NA	+	NA
27		46	F	0.1	NA	NA	+	NA
28		42	M	+	NA	renal cell carcinoma	+	NA
29		30	M	0.5	NA	myocardial infarction	+	NA
30		33	F	0.7	NA	NA	+	NA
31		32	M	1.0	NA	myocardial infarction	+	NA
32		20	M	+	NA	NA	+	NA
33	Sato et al.	23	M	2.0	0.9	NA	+	NA
34	Uesugi et al.	4	M	8.0	0.2	NA	+	NA
35		19	F	7.0	0.6	NA	NA	NA
36		27	M	1.0	0.8	NA	+	NA
37		58	M	12.0	1.6	NA	+	NA
38		75	M	2.1	NA	NA	+	NA
39	Niimi et al.	3	M	8.0	0.2	NA	+	NA
40	Castelletti et al.	59	F	NS	Nomal	NA	+	+
41		44	M	0.3	1.4	NA	+	+
42		40	M	NA	2.2	NA	+	+
43		35	M	NS	1.5	NA	+	+
44		18	M	4.2	1.4	NA	+	+
45		27	M	0.7	0.8	NA	+	+
46		16	M	NA	Nomal	NA	+	+
47		12	M	7.5	0.7	NA	+	+
48	Yong et al.	41	M	NS	1.7	NA	+	NA
49	Nadamuni et al.	50	F	4.8	0.8	anxiety, depression	+	+
50	Otsuka et al.	52	F	0.4	1.5	atrial septal defect tricuspid regurgitation	−	NA
51	Yoshino et al.	67	M	3.6	1.7	thymic carcinoid gastric cancer	−	NA
52	Baydar et al.	24	M	1.0	0.8	asthma	+	+
53	Ishimoto et al.	78	F	10.2	1.1	NA	−	−
54	Chen et al.	34	F	3.6	0.7	NA	−	NA
55		24	M	3.9	2.0	NA	−	NA
56		46	M	3.7	0.8	NA	+	NA
57		32	M	1.2	0.9	NA	−	NA
58		27	F	3.8	0.5	NA	−	NA
59		26	M	7.4	1.5	NA	−	NA
60		19	M	9.1	1.4	NA	−	NA
61		32	F	9.1	3.4	NA	−	NA
62		29	M	7.0	1.5	NA	−	NA
63		19	F	2.8	0.6	NA	−	NA
64	Ohtsubo et al.	20	M	0.86	92.1^a^	NA	+	+
65		13	F	1.6	115^a^	NA	−	+
66		35	F	ESRD	ESRD	NA	+	+
67		54	F	ESRD	ESRD	NA	+	+
68		35	F	5.85	76.3^a^	NA	−	+
69		15	F	0.51	86.5^a^	NA	+	+
70		53	F	11.8	48^a^	NA	−	+
71		16	M	0.44	124.1^a^	NA	−	+
72		74	M	3.56	63^a^	NA	−	+
73		38	F	4.9	55.3^a^	NA	+	+
74		14	F	10.1	123.4^a^	NA	−	+
75		64	M	4.6	46.3^a^	NA	−	+
76	This case	21	F	3.4	0.7	persistent cloaca congenital esophageal atresia	−	−

*F* female, *M* male, *UP* urinary protein (g/24 h), *Cre* creatinine (mg/dl), *NA* not available, *NS* nephrotic syndrome, *ESRD* end-stage renal disease

^a^eGFR (ml/min/1.73 m^2^)

Twelve of these 75 cases (16%) had some comorbidities, including two with renal cell carcinoma, but no cases had congenital malformations. The current patient developed fibronectin glomerulopathy, during the clinical course of persistent cloaca and congenital esophageal atresia. The direct pathophysiological relationship between fibronectin glomerulopathy and these congenital malformations remains unclear.

There have been numerous reports of cases with detailed family histories, but few reports of sporadic cases, such as the present patient [5, 19, 20, 22]. Eighteen of the 75 reported cases (24%) had no apparent family history of renal diseases. Furthermore, even in cases with a family history, the clinical courses were found to vary within families. For example, one patient progressed to ESRF, while another presented with no urine abnormalities [1, 15]. Ohtsubo et al. reported that some patients with *FN1* mutation were asymptomatic, and suggested that this genetic abnormality did not necessarily cause a phenotypic abnormality [8]. Furthermore, Castelletti et al. also reported a patient with fibronectin glomerulopathy and a genetic mutation, though the same mutation was not found in his family members, suggesting that it was a de novo mutation [2]. In the current case, there might have been asymptomatic patients with *FN1* mutations in her family, or the mutation might have arisen de novo. We did not perform renal biopsies and genetic tests in all her family members, and were therefore unable to establish the precise etiology of her disease.

Mutations at the *FN1* gene locus at 2q32 were first confirmed by Castelletti et al. in 2008 [2]. They identified three heterozygous missense mutations, Y973C, W1925R, and L1974R, but reported that these mutations only accounted for 40% of cases of fibronectin glomerulopathy [2]. Ohtsubo et al. subsequently identified six *FN1* mutations in 12 families with fibronectin glomerulopathy, including five novel *FN1* mutations (p.Pro969Leu, p.Pro1472del, p.Trp1925Cys, p.Lys1953_Ile1961del, and p.Leu1974Pro) [8]. However, none of these previously identified *FN1* mutations were detected in the current patient, despite genetic testing. This finding was in line with a previous report suggesting that some patients with fibronectin glomerulopathy showed no causative gene mutations in *FN1* [2, 5].

## Conclusion

We present a patient who developed fibronectin glomerulopathy, with no apparent familial history and no *FN1* mutation, during the clinical course of rare congenital malformations, including persistent cloaca and congenital esophageal atresia.

## Abbreviations

CH50: 50% hemolytic unit of complement
ESRF: End-stage renal failure
*FN1*: Fibronectin 1 gene
